# The Immune System’s Echo: The Phenomenon of Delayed Neurological Immune-Related Adverse Events (NirAEs) in Adjuvant Immunotherapy

**DOI:** 10.7759/cureus.44529

**Published:** 2023-09-01

**Authors:** Shruti Gohel, Zeid Kalarikkal, Viraj Lavingia, Jigar Mankad

**Affiliations:** 1 Medical Oncology, HealthCare Global (HCG) Cancer Center, Ahmedabad, Ahmedabad, IND; 2 Critical Care, Aurora St. Luke's Medical Center, Milwaukee, USA; 3 Neurology, Aurora St. Luke's Medical Center, Milwaukee, USA

**Keywords:** delayed immune-related adverse event (dire), immune-related adverse event (irae), meningoencephalitis, hypothyroidism, immunotherapy

## Abstract

The use of immune checkpoint inhibitors (ICIs) in early-stage settings has shown promise but can lead to chronic immune-related toxicities known as delayed immune-related adverse events (DIREs). These events, occurring after immunotherapy cessation, can affect various organ systems. Fatal immune-related adverse events (irAEs) are relatively rare but significant. Diagnostic challenges exist in distinguishing DIREs from disease sequelae. Efforts are needed to develop evidence-based strategies for managing DIREs as long-term survival with ICIs becomes possible. This case study highlights delayed neurological immune-related adverse events (NirAEs) encountered during pembrolizumab treatment, emphasizing the need for accurate diagnosis and prompt management. Reporting practices in immunotherapy trials hinder accurate assessment of DIREs. Close monitoring, accurate diagnosis, and timely corticosteroid administration are vital for effective DIRE management.

## Introduction

The promising new therapeutic applications of immune checkpoint inhibitors (ICIs) in early-stage settings have undoubtedly brought hope to patients and clinicians alike. However, emerging evidence has revealed a potential downside to these treatments: chronic immune-related toxicities affecting nearly 40% of the patients that are often overlooked and under-documented [1]. Couey et al. are credited with coining the term DIRE in 2019, which stands for delayed immune-related adverse events, defined as those manifesting ≥90 days after cessation of immunotherapy [2]. Chronic effects can affect various organ systems, including endocrine, rheumatological, pulmonary, and neurological adverse effects. The data gathered from multiple meta-analyses indicate that fatal immune-related adverse events (irAEs) are relatively rare but significant, with occurrence rates ranging from 0.4% when using anti-programmed cell death protein 1 (PD-1)/programmed death-ligand 1 (PD-L1) antibodies alone to approximately 1.2% when using a combination of anti-cytotoxic T-lymphocyte-associated antigen 4 (CTLA-4) and anti-PD-1 drugs [1]. The delayed occurrence of autoimmune events several months after the cessation of immunotherapy may be masqueraded as disease sequelae. This poses a diagnostic challenge as caution is needed in the analysis, grading, and appropriate interpretation of the literature to attribute them as immune-related adverse events. It is imperative that efforts are directed at developing more evidence-based strategies for managing delayed immune-related adverse events (irAEs), which becomes particularly relevant in the light of the possibility of projected long-term survival with ICIs.

## Case presentation

**Figure 1:**
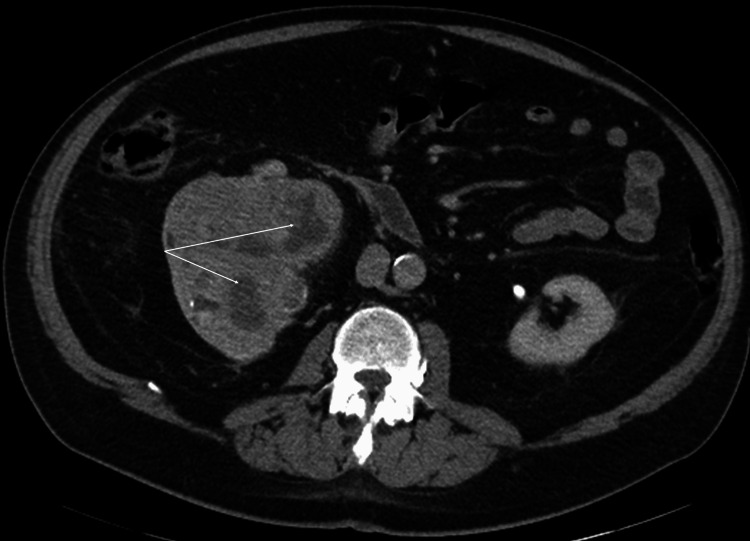
CT urogram CT urogram showed an 11 × 8.3 × 8.4 cm right kidney mass with an extension into the renal pelvis, concerning for aggressive renal cell carcinoma with extension into the renal pelvis or a renal pelvis urothelial carcinoma with extension toward the kidney parenchyma (arrows). CT: computed tomography

A 71-year-old male with a past medical history of hypertension, hyperlipidemia, atrial fibrillation, benign prostatic hyperplasia, obesity, and chronic kidney disease was diagnosed with kidney cancer when he experienced a sudden onset of gross hematuria. A computed tomography (CT) urogram revealed a heterogeneous 11 × 8.3 × 8.4 cm mass in the right kidney, which was highly concerning for renal cell carcinoma (RCC) or transitional cell cancer (TCC) (Figure 1).

**Figure 2:**
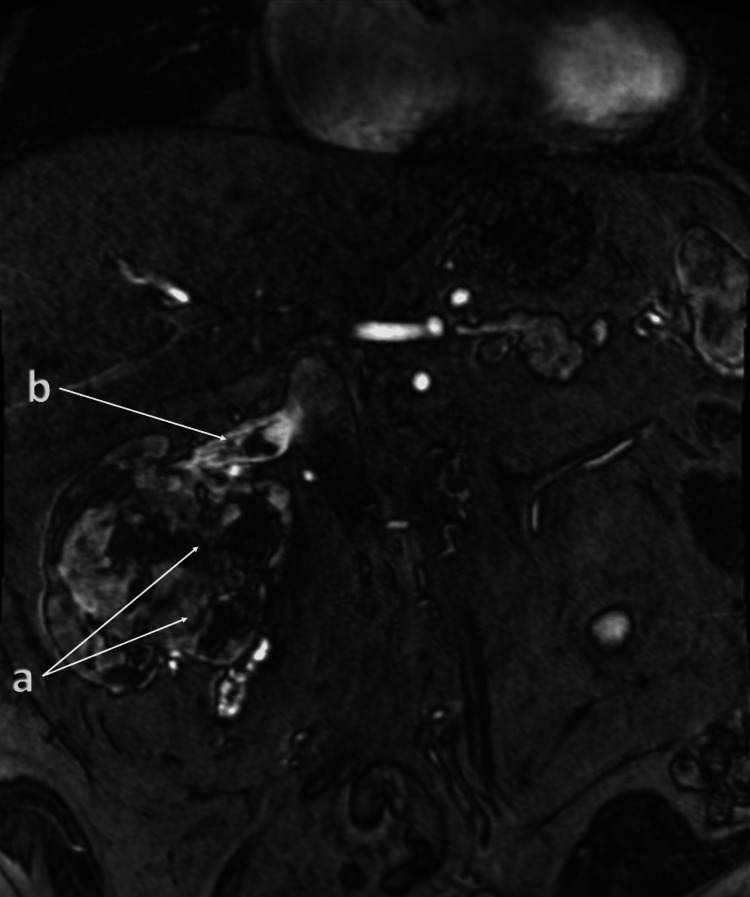
MRI angiogram of the abdomen MRI coronal image showed a large mixed solid and cystic mass (a, arrows) emanating from the right renal pelvis measuring 8.4 × 10.0 × 8.3 cm (AP × TR × CC). A focal filling defect (b, arrow) is seen within the draining right renal vein measuring approximately 2 cm long, demonstrating mild heterogeneous enhancement, and findings are most compatible with tumor thrombus. MRI: magnetic resonance imaging, AP: anteroposterior, TR: transverse, CC: craniocaudal

A chest CT with contrast showed no evidence of metastasis. A magnetic resonance imaging (MRI) angiogram of the abdomen showed a large mixed solid and cystic mass originating from the right renal pelvis and an expansile tumor thrombus within the right renal vein (Figure 2).

A bone scintigraphy study showed no definitive signs of metastatic disease. The patient subsequently underwent a robotic-assisted radical nephrectomy and renal vein tumor thrombectomy. Histopathology results confirmed grade 3 renal cell carcinoma with negative surgical margins and no lymph node involvement (pT3a, pN0) (Appendices).

**Figure 3:**
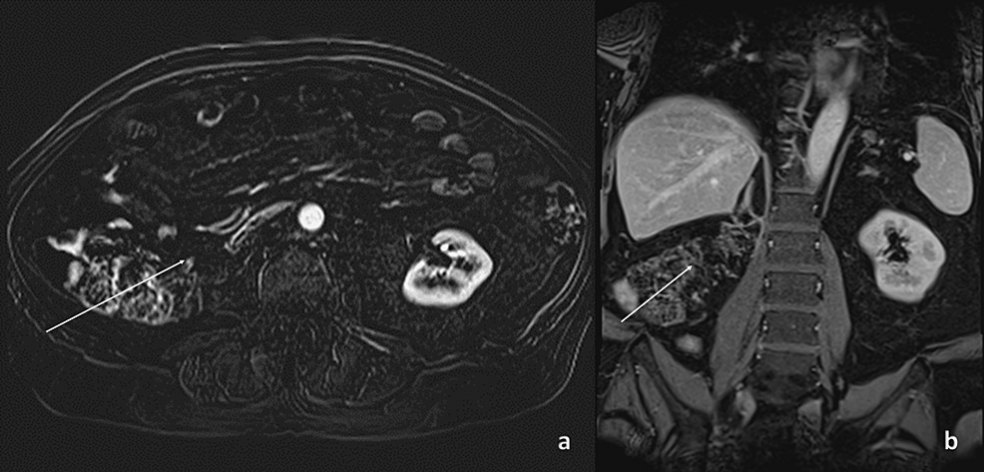
Follow-up MRI of the abdomen at three months after the initiation of therapy Post-radical right nephrectomy showed no convincing residual or recurrent tumor evident on axial (a, arrow) and coronal (b, arrow) sections. MRI: magnetic resonance imaging

To address the risk of recurrence, the patient was started on pembrolizumab (Keytruda) 200 mg intravenously every three weeks for 17 weeks, as initially planned. The patient tolerated the initial cycles well without experiencing fatigue, low blood counts, or endocrine imbalances. However, the patient had low folic acid levels and was prescribed supplements. Three months after starting immunotherapy with pembrolizumab, an MRI of the abdomen was conducted for follow-up, revealing no recurrence, residual tumor, or lymph node enlargement (Figure 3).

**Figure 4:**
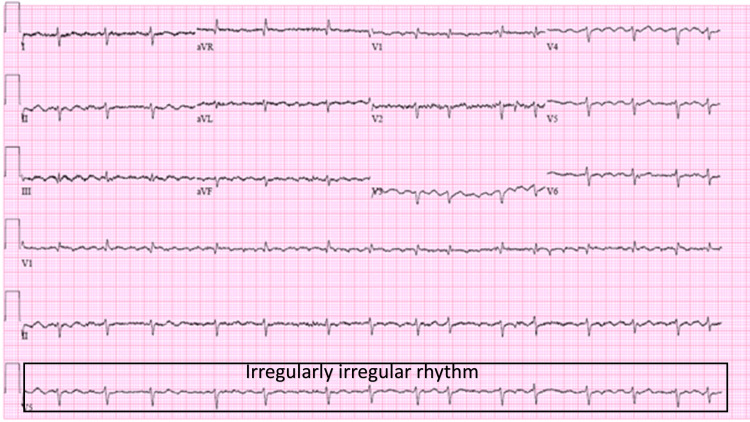
EKG showing atrial fibrillation EKG: electrocardiogram

During the course of treatment, the patient developed paroxysmal atrial fibrillation, as detected on an electrocardiogram (EKG) (Figure 4).

**Figure 5:**
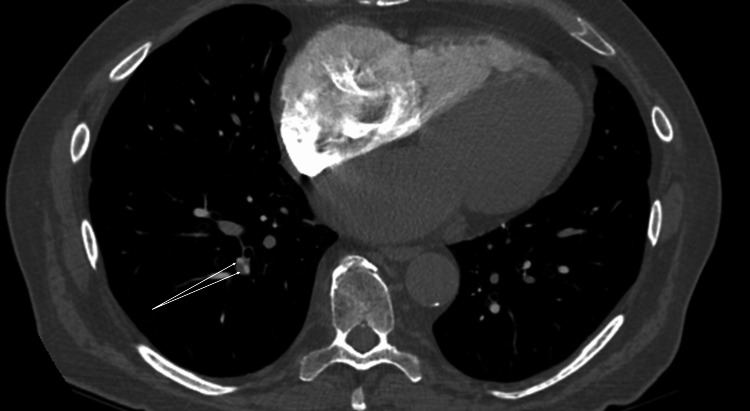
Chest CT PE protocol Chest CT shows a small filling defect on the right lower lung consistent with pulmonary arterial embolism (arrows). CT: computed tomography, PE: pulmonary embolism

The patient was referred to cardiology but was not initiated on anticoagulation therapy due to CHA2DS2-VASc (congestive heart failure, hypertension, age ≥ 75 years (doubled), diabetes mellitus, prior stroke or transient ischemic attack (TIA) or thromboembolism (doubled), vascular disease, age 65-74 years, and sex category) score of 1 and was managed with rate control therapy. The patient reported grade 1 fatigue, mild dizziness, and light-headedness during the ninth cycle of pembrolizumab, which worsened to grade 3 fatigue along with cytopenia by the 10th cycle. Consequently, pembrolizumab was discontinued, and a brain MRI was performed, which showed no acute process or evidence of intracranial metastatic disease. The patient’s white blood cell count (WBC) was 4.1 K/mcL, red blood cell count (RBC) was 3.87 million/mcL, hemoglobin (Hb) was 11.8 g/dL, hematocrit (Hct) was 35.6%, and platelet count was within the normal range at 233 K/mcL. Thyroid-stimulating hormone (TSH) levels at the 10th cycle were 1.954 mcUnits/mL, which decreased to 0.010 mcUnits/mL (normal value: 0.350-5.000 mcUnits/mL) during the two-week follow-up after the last pembrolizumab infusion. A repeat CT of the chest, abdomen, and pelvis showed no evidence of metastasis but identified a minor filling defect in a subsegmental branch of the pulmonary artery in the right lower lobe. A chest CT pulmonary embolism (PE) protocol confirmed subsegmental pulmonary embolism (Figure 5), and the patient was started on therapeutic anticoagulation.

**Figure 6:**
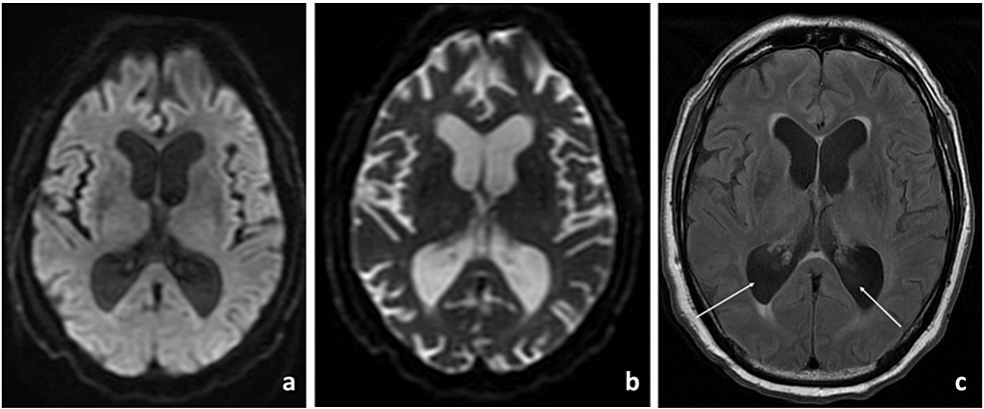
MRI of the brain MRI axial images showed no evidence of infarction on the diffusion-weighted image (a) and apparent diffusion coefficient (b) and showed enlarged ventricles with arrows on the fluid-attenuated inversion recovery image (c). MRI: magnetic resonance imaging

Due to persistent fatigue, the patient was prescribed dexamethasone 1 mg daily for 1-2 weeks and then transitioned to an oral prednisone taper over 6-8 weeks. Two weeks after completing the recommended steroid taper, the patient was admitted to the hospital with worsening fatigue, shortness of breath, and progressive leg swelling for 1-2 weeks. Sialadenitis or angioedema was suspected due to concerns about airway compromise. Nasotracheal intubation was attempted but failed due to epistaxis, and the patient was eventually intubated orotracheally. Subsequently, the patient experienced cardiac arrest with pulseless electrical activity (PEA) and was resuscitated after 13 minutes. Toward the end of the first week of hospitalization, the patient experienced a sudden drop in hemoglobin and was diagnosed with hemorrhagic shock secondary to a spontaneous retroperitoneal hematoma, necessitating transfer to a higher level of care for interventional radiology (IR)-guided intervention. Exploratory laparotomy was performed, and continuous renal replacement therapy was required. Two weeks after the initial admission, the patient was extubated, and an inferior vena cava (IVC) filter was placed due to the inability to anticoagulate the patient. The patient experienced periods of lethargy and increased muscle tone. Thyroid function tests revealed severe hypothyroidism, and an endocrine specialist was involved in the patient’s care. The appropriate thyroid supplementation therapy was initiated. Despite correcting the underlying hypothyroidism, the patient’s lethargy, mental status changes, and increased muscle tone worsened, leading to repeat intubation. A brain MRI showed no acute process but revealed relatively enlarged ventricles, raising concern for normal pressure hydrocephalus (Figure 6).

Neurology service was consulted, and a lumbar puncture was recommended to rule out any infectious process, which showed elevated protein levels of 137 mg/dL and 49 total nucleated cells in the cerebrospinal fluid (CSF). Extensive CSF analysis, including syphilis screening (Venereal Disease Research Laboratory (VDRL)), meningitis panel, encephalitis panel, cryptococcal antigen, and fungal culture, all returned negative. The infectious disease team was also involved in the patient’s care and agreed that the CSF findings were inconsistent with an underlying infectious process. Serum workups, including glutamic acid decarboxylase (GAD) antibody, Mayo Clinic autoimmune dysautonomia panel, Mayo Clinic paraneoplastic panel, anti-glycine receptor antibody, and N-methyl D-aspartate (NMDA) receptor antibody tests, were all negative. A CSF encephalitis panel was sent to the Mayo Clinic, which also returned negative. Eventually, the patient was started on high-dose intravenous methylprednisolone for five days and underwent plasma exchange every other day for five cycles. The patient’s mental status slowly improved but required a tracheostomy due to prolonged ventilatory support. The patient was eventually diagnosed with immune checkpoint inhibitor (pembrolizumab)-related encephalitis and initiated on a prednisone taper over 4-6 months. The patient was transferred to a rehabilitation facility, where the tracheostomy was closed, and he made good progress with physical and speech therapy. Four months later, the patient was discharged home and reported feeling nearly back to himself during outpatient neurology follow-up, with only mild residual right quadriceps weakness, believed to be related to a peripheral nerve issue.

## Discussion

Rationale/underlying mechanism

Multiple studies have provided evidence supporting the idea that immunotherapy has lasting effects beyond the withdrawal of immune checkpoint blockades (ICBs), potentially due to the development of “immunological memory.” If this phenomenon is associated with positive outcomes, it could also have important implications for managing T cell-mediated toxicities, which can manifest long after the discontinuation of immunotherapy [1].

The occupation of the PD-1 receptor on T cells reaches a maximum of 80%-90% after a single dose of nivolumab, and this level is maintained for up to 90 days, with the mean PD-1 receptor occupancy on T cells plateauing at 72% for a period of ≤57 days, despite the serum half-life of the drug being only 12-20 days [2]. After three doses, the occupation remains at 40% for over eight months after the last dose. These findings imply that the sustained pharmacodynamic effects of ICIs typically exceed their pharmacokinetic half-life. Similarly, just as the sustained clinical response after immunotherapy seems to be unrelated to the dose and length of the treatment, immune-related toxicity may also have a varying onset with the immune treatment [3].

NirAEs

Among the possible long-term effects, neurological immune-related adverse events (NirAEs) have become a growing concern, affecting up to 5% of patients receiving immunotherapy, as they can be persistent and severe [4]. The impacts of NirAEs can be bleak, affecting the neuromuscular junction, central nervous system, and sensory and motor peripheral nerves, manifesting as myasthenia gravis and Lambert-Eaton myasthenic syndrome (LEMS), meningoencephalitis, and Guillain-Barré syndrome (GBS), respectively. Among these events, the peripheral nervous system is affected approximately twice as often as the central nervous system [5].

According to a study, patients who received ICIs had a higher incidence of certain neurological adverse events than the general population. Specifically, myasthenia gravis, encephalitis, peripheral neuropathy, and meningitis were more commonly reported in patients receiving ICIs. Myasthenia gravis and encephalitis were more likely to be associated with anti-PD-1 ICIs, while other neurological adverse events were more commonly associated with anti-CTLA-4 ICIs. Myasthenia gravis was particularly concerning, as it had a high fatality rate of approximately 20% and tended to occur early in treatment (median: 29 days). It often occurs in conjunction with myocarditis and myositis, and its long-term outcomes are not well-defined. Guillain-Barré syndrome (GBS) has a high fatality rate and often leaves residual symptoms even after treatment. In one series of 31 patients, approximately one in five patients with GBS died, and 68% of patients had residual symptoms after treatment [2].

Other neurological adverse events had lower fatality rates of 6%-12%, tended to occur later in treatment (median: 61-80 days), and were not typically associated with other concurrent neurological conditions [6]. Meningoencephalitis can be resolved with appropriate acute management, but chronic deficits caused by it remain uncertain.

Peripheral neuropathy is a chronic irAE associated with smoldering inflammation caused by ICIs. In one study, 2% of resected melanoma patients experienced peripheral sensory neuropathy after receiving anti-PD-1 antibodies in the adjuvant setting, and half of them developed chronic sensory neuropathy [7]. This can be particularly challenging for patients as the symptoms can affect their basic activities of daily living (ADLs) and can be debilitating. As with any treatment, the benefits and risks of ICIs must be carefully weighed, particularly when considering the possibility of long-term survival. Understanding the potential toxicities of these treatments is crucial in providing the best care for patients and advancing the field of cancer immunotherapy.

Challenges

There are challenges in determining the actual frequency and extent of DIRE due to limitations in current reporting practices in immunotherapy clinical trials. Problems include short follow-up periods and incomplete reporting of irAEs [8]. Some trial protocols specifically exclude reporting adverse events that occur more than four weeks after the last dose or after starting another cancer-directed therapy. Additionally, irAEs are usually reported based on the start of treatment but do not include information about the previous amount of immunotherapy [9]. This indicates a potential diagnostic challenge in the curative approaches expected to become more prevalent with the approval of new immunotherapy treatments in neoadjuvant and adjuvant settings. A recent retrospective analysis indicated that chronic immune-related adverse events (irAEs) lasting more than 12 weeks after discontinuing an anti-PD-1/PD-L1 antibody were more frequent than previously reported. The study found that 43.2% of patients experienced chronic irAEs [7].

Limitations

There is a paucity of research on delayed immune-related adverse events (DIREs) following the cessation of immunotherapy, primarily due to insufficient follow-up durations in most studies. The direct link between immunotherapy and delayed autoimmune reactions is not established, especially with case reports, making it difficult to determine a cause-and-effect relationship [10,11]. Case reports regarding diagnosis and treatment vary in detail, but with increased awareness, more emphasis will be placed on diagnostic methods and treatment strategies. Determining the actual frequency and extent of delayed autoimmune reactions is challenging due to limitations in clinical trial reporting practices that only provide information relative to the start of treatment.

## Conclusions

Clinical understanding of delayed immune-related adverse events (DIREs) can prevent potential harm and mortality from misdiagnoses and unnecessary diagnostic and treatment interventions. Prompt and effective management of DIREs through immunosuppressive therapy is possible. Incorrect diagnosis of DIREs and NirAEs poses threats, including invasive procedures, e.g., Ommaya reservoir placement and lumbar puncture. It may also lead to sometimes mistakenly discontinuing potentially beneficial cancer-directed therapy. In clinical research, encountering a conundrum where symptoms or clinical observations are erroneously attributed to coexisting factors such as chemotherapy, radiation therapy, disease relapse, or septicemia is not uncommon. These diagnostic errors can potentially introduce confounding factors that challenge the accuracy and reliability of research outcomes. Additionally, we aimed to explore if nomograms, such as the Naranjo algorithm, can assist clinicians in making informed decisions regarding managing DIREs and aid in monitoring and reporting adverse events in immunotherapy. The Naranjo algorithm evaluates various criteria, including the temporal relationship between the onset of symptoms and immunotherapy, previous exposure to similar drugs, and the resolution of symptoms upon drug discontinuation or rechallenge. Close monitoring of patients, prompt diagnoses, and timely administration of corticosteroids are crucial steps for effectively managing the condition.

## Appendices

Appendix 1: Pathology report

Pathologic Diagnosis

A. Lymph node, aortocaval, biopsy: single small benign lymph node, negative for metastatic carcinoma (0/1).

B. Kidney, right, radical nephrectomy: clear-cell renal cell carcinoma, grade 3 (of 4), forming a partially necrotic 9.5 cm mass extending into the renal vein and renal sinus fat; all surgical resection margins are negative for malignancy; and AJCC stage pT3a, pN0.

C. Liver, wedge biopsy: benign hepatic parenchyma, negative for malignancy.

Gross Description

A. Received in formalin, labeled with the patient’s barcode and “intro aortocaval node,” is a 2.5 × 2.0 × 0.8 cm portion of fragmented pink to yellow rubbery tissue, which is bisected and entirely submitted in two cassettes.

B. Specimen labeled: Properly labeled with the patient’s barcode and “right radical nephrectomy”

Fixative: Fresh

Specimen received: Nephrectomy

Overall dimensions (to include fat): 32.0 × 13.5 × 12.0 cm

Ureter: Patent, 9.5 cm in length

Renal artery and vein: Renal arteries are widely patent. The renal vein is patent at the margin, 0.6 cm from the resection margin; a recognizable tumor thrombus protrudes into the lumen.

Adipose integrity: Intact, abundant

Kidney dimensions: 15.0 × 9.0 × 9.0 cm

Cortical surface: Predominantly brown and smooth with some yellow discoloration

Gross findings: Sectioning reveals an ill-defined, multilobulated, nonencapsulated 9.5 × 9.0 × 9.0 cm yellow-orange to brown-gray mass obliterating the mid- and lower poles. The mass is identified within all poles, extending into the renal sinus and venous structures (location: the mass is identified within all poles, distance to margins: the mass is 0.6 cm from the Gerota’s fascia, 0.6 cm from the hilum, and 0.15 cm from the blue-inked capsule).

Remaining cut surfaces: Tan to red-brown with poorly demarcated cortical medullary junctions

Weight: 725 g

Adrenal gland: No adrenal gland is identified.

Lymph nodes: No lymph nodes are identified

Representative sections: 1: ureteral and vascular margins en face, cut toward the true margin, 2: mass with adjacent renal sinus, 3: mass with pelvic mucosa, 4: mass within the renal vein, 5-6: mass with inked Gerota’s fascia, 7: mass to the renal capsule, 8-9: random mass, 10: random remaining kidney

C. Received in formalin, labeled with the patient’s barcode and “liver biopsy,” is a 0.6 × 0.4 × 0.4 cm cauterized tan to red-brown rubbery tissue submitted in toto in one cassette.

Microscopic Description

A. Microscopic findings support the above diagnosis.

B. See synoptic report.

Selected slides containing the tumor (blocks B2, B3, B4, and B9) were reviewed in consultation with a pathologist, who concurred with the type and grade of the neoplasm.

C. Microscopic findings support the above diagnosis.
